# Widely Targeted Lipidomics of Nasal Fluid Across Upper-Airway Inflammation: Phenotype-Associated Remodeling in Allergic Rhinitis and Chronic Rhinosinusitis with Nasal Polyps

**DOI:** 10.3390/ijms27219715

**Published:** 2026-10-29

**Authors:** Yuzhuo Liu, Jinbo Han, Xiaowei Wang, Weiqing Wang, Lei Wang, Yang Zha, Wei Lv, Zhenzhen Zhu

**Affiliations:** 1 Department of Otolaryngology-Head and Neck Surgery, Peking Union Medical College Hospital, Chinese Academy of Medical Sciences & Peking Union Medical College, Beijing 100730, China; lyzminminliu@163.com (Y.L.); adnataster@163.com (J.H.); 13701397599@163.com (X.W.); weiqing.wang@outlook.com (W.W.); wanglei_199212@126.com (L.W.); zhayang@live.cn (Y.Z.); 2 Department of Otolaryngology-Head and Neck Surgery, Beijing Chao-Yang Hospital, Capital Medical University, Beijing 100730, China

**Keywords:** nasal fluid, lipidomics, chronic rhinosinusitis with nasal polyps, allergic rhinitis, eosinophilic inflammation, lipid remodeling

## Abstract

Allergic rhinitis (AR) and chronic rhinosinusitis with nasal polyps (CRSwNP) share overlapping inflammatory mechanisms while retaining distinct disease biology. Although lipid metabolism is increasingly implicated in airway inflammation, the nasal fluid lipidomic landscape across AR and CRSwNP and its relationship to local eosinophilic inflammation and clinical burden remain incompletely understood. We performed widely targeted liquid chromatography–tandem mass spectrometry (LC-MS/MS) lipidomics on nasal fluid from 73 participants, including 22 controls, 23 patients with AR, and 28 patients with CRSwNP. Group differences were evaluated using multivariate analysis and models adjusted for age, sex, and smoking status, followed by trajectory clustering and residual-correlation network analysis. Within CRSwNP, adjusted models were used to assess lipid associations with tissue eosinophilia, the 22-item Sinonasal Outcome Test (SNOT-22), overall visual analog scale (VAS), and Lund–Mackay computed tomography score. Lipid subclass patterns were examined by ranked enrichment and focused subclass score analyses. AR was characterized mainly by higher relative abundances of TG species, whereas CRSwNP showed broader increases involving triacylglycerol species, plasmalogen phosphatidylethanolamines, and lysophospholipids. Exploratory clustering of 147 lipids showing nominally significant differences (*p* < 0.05) in at least one covariate-adjusted pairwise group comparison yielded four modules with differing biochemical compositions, although the partition was sensitive to the input lipid set. Among these, the diacylglycerol–lysophosphatidic acid-related module displayed strong internal network connectivity, suggesting coordinated variation within the identified module. Within CRSwNP, ranked analysis of lipid associations with tissue eosinophilia showed negative enrichment of plasmalogen phosphatidylethanolamine, sphingomyelin, and ether-linked lysophosphatidylcholine, and positive enrichment of glycerolipid and acylcarnitine subclasses. Clinical burden showed only partial overlap with this pattern: plasmalogen phosphatidylethanolamine was inversely associated with both tissue eosinophilia and patient-reported symptom burden, whereas conventional lysophosphatidylcholine showed stronger positive associations with symptomatic and radiographic burden. These findings suggest distinct and coordinated nasal fluid lipidomic features associated with the upper-airway inflammatory phenotype, local eosinophilic inflammation, and clinical disease burden.

## 1. Introduction

Allergic rhinitis (AR) and chronic rhinosinusitis with nasal polyps (CRSwNP) are common chronic inflammatory disorders of the upper airway. The well-recognized association between the two conditions is likely attributable to shared mucosal inflammatory mechanisms, including epithelial barrier dysfunction, IgE-associated immune activation and eosinophilic immune responses [1,2,3]. Despite their frequent coexistence, AR and CRSwNP represent distinct inflammatory diseases rather than successive stages along a common pathological continuum. Their pathophysiological overlap is most evident in eosinophilic CRSwNP, whereas other CRSwNP endotypes exhibit distinct or mixed inflammatory programs [1,4]. Defining the shared and disease-specific molecular programs of AR and CRSwNP may therefore provide a biological framework for understanding their frequent co-occurrence and distinct clinical phenotypes.

Lipid metabolism is closely integrated with mucosal homeostasis and inflammation [5,6]. Functionally, lipids can be viewed as membrane structural components, metabolic and energy storage substrates, and bioactive signaling molecules. Through these interconnected roles, lipid metabolism influences membrane organization, cellular energetics, immune signaling, and both the initiation and resolution of inflammation [5,6,7,8]. Dysregulation of phospholipids, sphingolipids, fatty acids, and mediators derived from polyunsaturated fatty acids (PUFAs) has been increasingly recognized across inflammatory airway diseases [8,9,10,11]. In AR, lipid mediators are integral to the local allergic response. Nasal allergen challenge induces rapid release of mast-cell-associated prostaglandin D2 (PGD2) and cysteinyl leukotrienes such as cysteinyl leukotriene C4 (LTC4) into nasal secretions, in parallel with the development of characteristic early-phase symptoms of sneezing, rhinorrhea, and nasal obstruction [12,13]. More recent metabolomic and lipidomic studies have extended these observations beyond classical inflammatory mediators, identifying alterations in fatty-acid, glycerophospholipid, and sphingolipid metabolism. Sphingosine-1-phosphate, linoleic acid, diacylglycerol (DG), triacylglycerol (TG), and lysophosphatidylcholine (LPC) have been linked to symptom burden [14,15] or inflammatory markers such as IgE and IL-33 [16]. In CRS, lipidomic alterations appear to reflect both the inflammatory phenotype and the balance between inflammatory activation and resolution [17,18]. Tissue lipidomic and metabolomic studies have identified broad changes in lipid composition and metabolism, with distinct metabolic patterns across CRS inflammatory endotypes [18,19]. In particular, eosinophilic CRSwNP shows enhanced unsaturated fatty acid oxidation that is associated with tissue eosinophilia and IL-5 expression [18]. Lipid mediators that promote the resolution of inflammation are also altered in CRS, including maresins [20], resolvins, and lipoxins [17]. In CRSwNP, altered maresin concentrations in nasal secretions and plasma have further been associated with quality-of-life measures [20]. Membrane lipid remodeling provides a complementary perspective on these inflammatory processes. Plasmalogens are ether glycerophospholipids characterized by a vinyl-ether linkage at sn-1 and an ester-linked acyl chain at sn-2. They contribute to membrane organization and may limit lipid oxidation through reactions involving the vinyl–ether bond, although their antioxidant activity is context-dependent [21]. This linkage also renders them susceptible to oxidative cleavage, including attack by eosinophil peroxidase-derived brominating species [22]. Sphingolipid metabolism connects structural sphingomyelin with bioactive ceramide and related signaling lipids, linking membrane remodeling to inflammatory and cellular stress responses [23]. These properties provide a biological rationale for examining plasmalogen and sphingolipid remodeling in upper-airway inflammation. Nasal fluid provides an accessible compartment for examining the local sinonasal inflammatory microenvironment. Its cellular and cytokine profiles differ between eosinophilic and non-eosinophilic CRSwNP, and selected cytokines correlate between paired nasal secretions and sinus tissue [24,25]. Lipid alterations have also been documented in sinus secretions from patients with CRS [26]. Nevertheless, the broader lipidomic landscape of nasal fluid across AR and CRSwNP and its relationship to the local inflammatory phenotype and clinical burden remain insufficiently characterized.

In this study, we profiled the nasal fluid lipidome across controls, AR, and CRSwNP to characterize shared and disease-specific lipid remodeling and its coordinated organization across disease states. Within CRSwNP, we further investigated how lipid species and structural subclasses were associated with tissue eosinophilia and clinical burden. We compared these associations to identify shared and distinct lipidomic patterns between disease phenotypes.

## 2. Results

### 2.1. Nasal Fluid Lipidomic Profiling Across Clinical Groups

**Figure 1:**
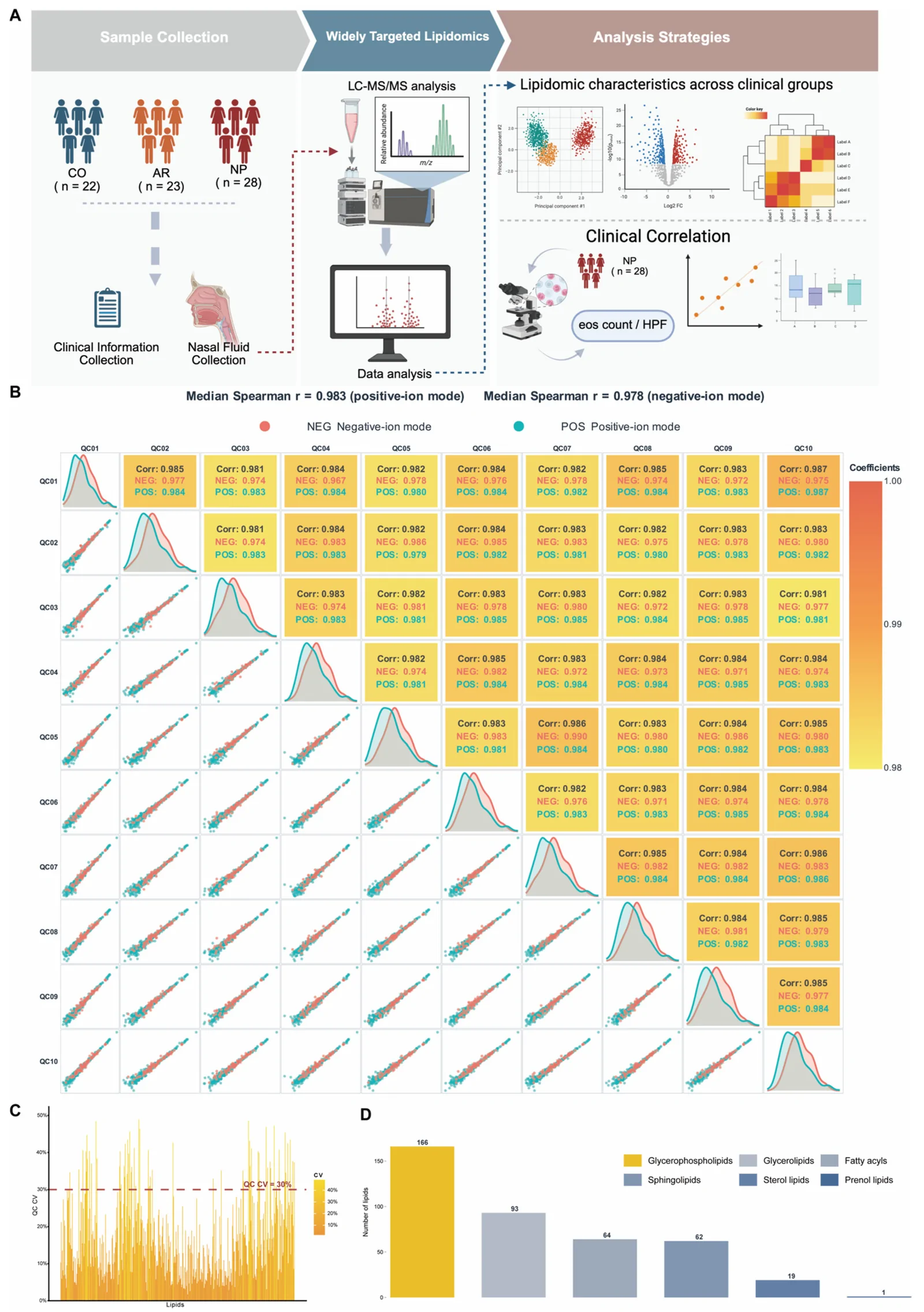
Study design and analytical quality control. (**A**) Study overview. Nasal fluid was collected from 73 participants classified as controls (CO; *n* = 22), allergic rhinitis (AR; *n* = 23), or chronic rhinosinusitis with nasal polyps (NP; *n* = 28) and analyzed by widely targeted UPLC–ESI–MS/MS lipidomics. Tissue eosinophilia and clinical-burden analyses were subsequently performed within the NP group; tissue eosinophil measurements were available for 27 participants. (**B**) Pairwise Spearman correlations among pooled quality-control (QC) samples in positive- and negative-ion modes. (**C**) Distribution of QC coefficients of variation (CVs) across the 465 quantified lipids, with the 30% retention threshold indicated. (**D**) Lipid-category composition of the 405 lipids retained for downstream analyses.

The study included 73 participants, comprising 22 controls (CO), 23 patients with allergic rhinitis (AR), and 28 patients with chronic rhinosinusitis with nasal polyps (NP) (Figure 1A). Median age was higher in NP (49.0 [33.8, 55.2] years) than in CO (32.0 [27.0, 44.5] years) or AR (31.0 [27.0, 40.0] years; *p* = 0.004), while sex (*p* = 0.040) and smoking status (*p* = 0.037) also differed across groups. Blood eosinophil percentage was higher across the inflammatory phenotypes (CO, 2.2 [1.2, 2.9]%; AR, 3.8 [2.5, 5.0]%; NP, 5.0 [3.1, 8.6]%; *p* = 0.006). The median overall sinonasal symptom VAS score was 6.0 [5.5, 7.0] in the AR group and 7.0 [6.0, 7.5] in the NP group, with no statistically significant difference between the two study groups (*p* = 0.372). In the NP group, the median SNOT-22 score was 37.0 [21.0, 49.0] and the median Lund–Mackay CT score was 20.0 [16.0, 22.0] (Table 1). Age, sex, and smoking status were included as covariates in subsequent adjusted analyses.

Widely targeted LC–MS/MS profiling quantified 465 nasal-fluid lipids, including 392 in positive-ion mode and 73 in negative-ion mode. The median pairwise Spearman correlation among pooled-QC samples was 0.983 in positive-ion mode and 0.978 in negative-ion mode (Figure 1B). After applying the 30% QC-CV threshold, 405 lipids were retained for downstream analyses (Figure 1C). These comprised 166 glycerophospholipids, 93 glycerolipids, 64 fatty acyls, 62 sphingolipids, 19 sterol lipids, and 1 prenol lipid (Figure 1D).

### 2.2. Distinct but Partially Overlapping Nasal Fluid Lipidomic Profiles Across CO, AR, and NP

**Figure 2:**
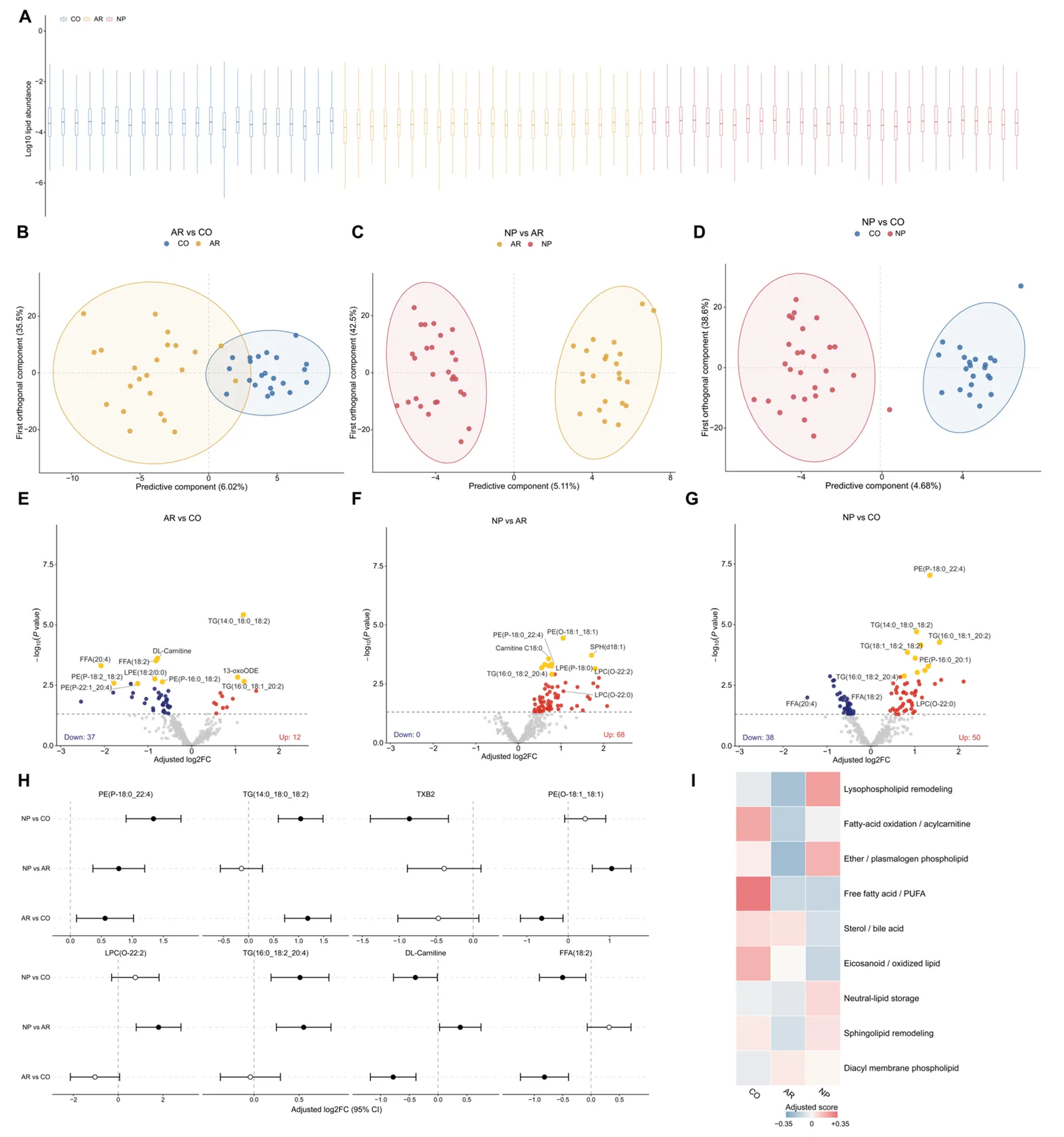
Nasal fluid lipidomic differences across CO, AR, and NP. (**A**) Sample-wise distributions of log10-transformed, total-peak-area-normalized relative lipid abundance across controls (CO), allergic rhinitis (AR), and chronic rhinosinusitis with nasal polyps (NP). (**B**–**D**) Pairwise OPLS-DA score plots for AR versus CO, NP versus AR, and NP versus CO, respectively. For AR-CO, NP-AR, and NP-CO, R^2^X/R^2^Y/Q^2^ were 0.550/0.778/0.318, 0.644/0.951/0.501, and 0.559/0.928/0.644, respectively. Permutation *p* values for Q^2^ were 0.005 in all three models, while those for R^2^Y were 0.100, 0.010, and 0.005, respectively (200 permutations; Supplementary Figure S3 and Supplementary Table S2). (**E**–**G**) Volcano plots showing age-, sex-, and smoking-adjusted lipid differences for the corresponding pairwise comparisons; labeled lipids highlight representative changes. Volcano plots display nominal *p* values from covariate-adjusted models; corresponding BH-adjusted *p* values are provided in Supplementary Table S2. Blue and red points indicate lipids with negative and positive adjusted log2 fold changes at nominal *p* < 0.05 respectively; gray points indicate lipids with *p* ≥ 0.05, and yellow points highlight the top 10 lipids ranked by *p* value in each pairwise comparison. (**H**) Adjusted log2 fold changes and 95% confidence intervals for eight representative lipids. (**I**) Covariate-adjusted mean scores of nine predefined structural–functional lipid programs across CO, AR, and NP. Pairwise score differences and 95% confidence intervals are shown in Supplementary Figure S2.

The sample-wise distributions of log10-transformed lipid abundance after total-peak-area normalization are shown in Figure 2A. PCA of all 405 retained lipids is shown in Supplementary Figure S1. Pairwise OPLS-DA showed clearer separation in comparisons involving NP, whereas separation between AR and CO was less distinct (Figure 2B–D).

Covariate-adjusted analyses revealed different patterns of lipid change across the three comparisons (Figure 2E–G). At *p* < 0.05, 49, 68, and 88 lipid species differed in AR versus CO, NP versus AR, and NP versus CO, respectively. After BH correction, 3, 11, and 12 species, respectively, remained significant at BH-adjusted *p* < 0.05. The broader nominal-P patterns were retained for exploratory characterization (Supplementary Table S2). Relative to CO, AR was characterized predominantly by lower free fatty acid (FFA), free carnitine, and several ether/plasmalogen phospholipids, together with higher abundance of selected TG species (Figure 2E). In contrast, NP showed broad increases relative to AR across ether- and plasmalogen-containing phospholipids, ether-linked LPC species, sphingolipid-related species, acylcarnitines, and TG species (Figure 2F). Compared with CO, NP exhibited a mixed pattern, with higher plasmalogen phosphatidylethanolamines (PE) and selected TG species but lower FFA, free carnitine, and thromboxane-related species (Figure 2G). Among the 64 participants without asthma, all originally nominally significant between-group lipid differences retained their direction, although none in the NP-AR comparison met BH-adjusted *p* < 0.05 (Supplementary Table S5).

Representative lipids further illustrated these distinct cross-group patterns (Figure 2H). PE(P-18:0_22:4), a plasmalogen PE, increased from CO through AR to NP, whereas TG(14:0_18:0_18:2) was similarly elevated in both AR and NP relative to CO. The ether phospholipids PE(O-18:1_18:1) and LPC(O-22:2) showed an AR-low pattern with higher levels in NP, while the PUFA-containing TG(16:0_18:2_20:4) was preferentially increased in NP. Conversely, DL-carnitine and FFA(18:2) were lower in both inflammatory groups than in CO, and TXB2 was lower in NP than in CO.

To capture coordinated lipid changes beyond individual species, we summarized the retained lipids into nine predefined structural–functional programs representing major dimensions of membrane lipid remodeling, fatty acid handling and oxidation, neutral lipid storage, sphingolipid remodeling, sterol/bile acid metabolism, and eicosanoid/oxidized lipid biology. These programs showed distinct adjusted patterns across CO, AR, and NP (Figure 2I). NP was characterized by relatively higher lysophospholipid remodeling, ether/plasmalogen phospholipid, and neutral lipid storage scores, together with lower free fatty acid/PUFA and eicosanoid/oxidized lipid scores. In contrast, AR showed comparatively lower fatty acid oxidation/acylcarnitine, ether/plasmalogen phospholipid, and sphingolipid remodeling scores. These coordinated program-level differences were consistent with the species-level patterns and further supported phenotype-associated differences in relative lipid composition. Pairwise program-score comparisons showed nominal differences, but none remained significant after BH correction (Supplementary Figure S2).

### 2.3. Phenotype-Associated Lipids Organize into Coordinated Cross-Group Modules

**Figure 3:**
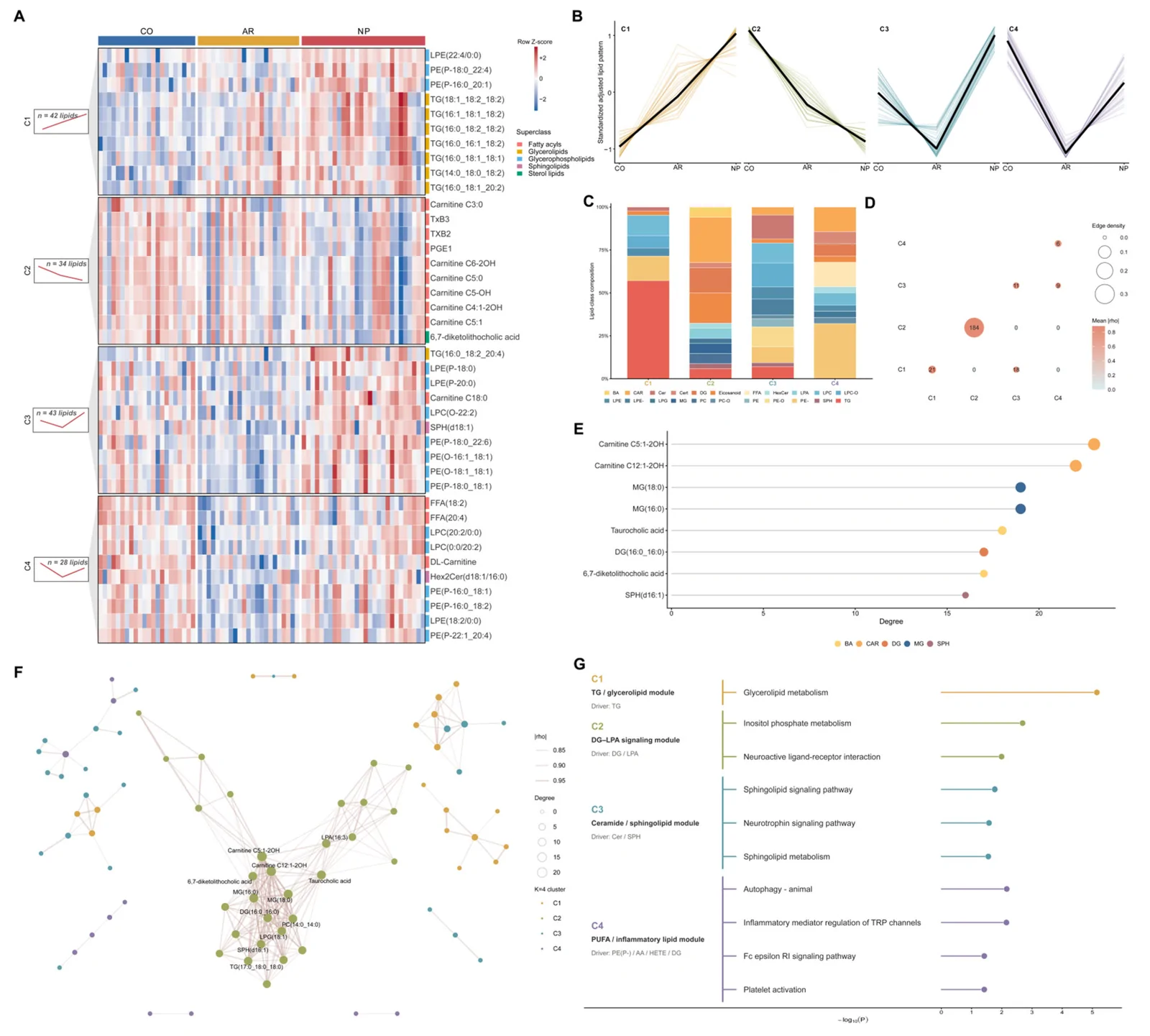
Cross-group lipid modules and residual-correlation network structure. (**A**) Heatmap of 40 representative lipids (10 per module) from the 147 candidate lipids meeting nominal *p* < 0.05 in at least one adjusted pairwise comparison, grouped into four exploratory K-means modules across CO, AR, and NP. The n values indicate total module membership. (**B**) Standardized cross-group abundance patterns of the four modules. (**C**) Lipid-class composition of each module. (**D**) Distribution of residual lipid–lipid correlations within and between modules; circle size represents edge density, and color indicates mean absolute correlation. (**E**) Top network hub lipids ranked by node degree in the residual-correlation network. (**F**) Residual Spearman correlation network colored by module membership, with node color indicating module membership and node size indicating degree. (**G**) Cluster-specific KEGG pathway enrichment.

The 147 lipids meeting *p* < 0.05 in at least one covariate-adjusted pairwise group comparison were organized into four exploratory modules with distinct cross-group abundance patterns and lipid compositions (Figure 3A–C). C1 (42 lipids) showed an overall CO < AR < NP pattern and contained many TG species, together with plasmalogen PE and lysophospholipid species. C2 (34 lipids) showed the opposite NP < AR < CO pattern and included acylcarnitines, eicosanoid-related lipids, and DG species. C3 (43 lipids) followed an overall AR < CO < NP pattern and was characterized by ether/plasmalogen phospholipids, LPC/lysophosphatidylethanolamine (LPE) species, Cer species, and other sphingolipids. C4 (28 lipids) showed an overall AR < NP < CO pattern and included plasmalogen PE, FFA, and acylcarnitines.

The residual correlation analysis revealed a network structure that broadly reflected the K-means partition of the 147 candidate lipids. Among the 249 strong lipid–lipid correlations, 222 (89.2%) occurred within the same module, significantly more than expected under random module assignment (permutation *p* = 1.0 × 10^−4^) (Figure 3D,F). Notably, network connectivity was strongly concentrated in C2, the NP < AR < CO module, with 184 C2-C2 connections forming the dominant densely connected component. The highest-degree nodes were all assigned to C2, including Carnitine C5:1-2OH (degree = 23) and Carnitine C12:1-2OH (degree = 22), together with MG and DG species, bile acid-related lipids, and SPH(d16:1) (Figure 3E). Across the three thresholds, within-module edges accounted for 80.9–92.0% of all edges, and C2 contained the largest number of within-module edges at every threshold. Individual hub rankings varied, and C4 had no within-module edges at the strictest threshold (Supplementary Table S3).

Exploratory KEGG analysis was used to examine pathway associations within each module (Figure 3G). The TG-rich C1 module showed a glycerolipid metabolism signature. The DG-LPA-related C2 module was associated with inositol phosphate and receptor-mediated signaling terms, whereas C3 showed enrichment related to sphingolipid metabolism and signaling, together with neurotrophin-related processes. C4 annotations included TRP channel regulation, FcεRI signaling, autophagy, and platelet activation. These associations provide putative biological context for the modules rather than evidence of pathway activation.

### 2.4. Tissue Eosinophilia Is Associated with Coordinated Lipid-Subclass Remodeling Within the NP Group

**Figure 4:**
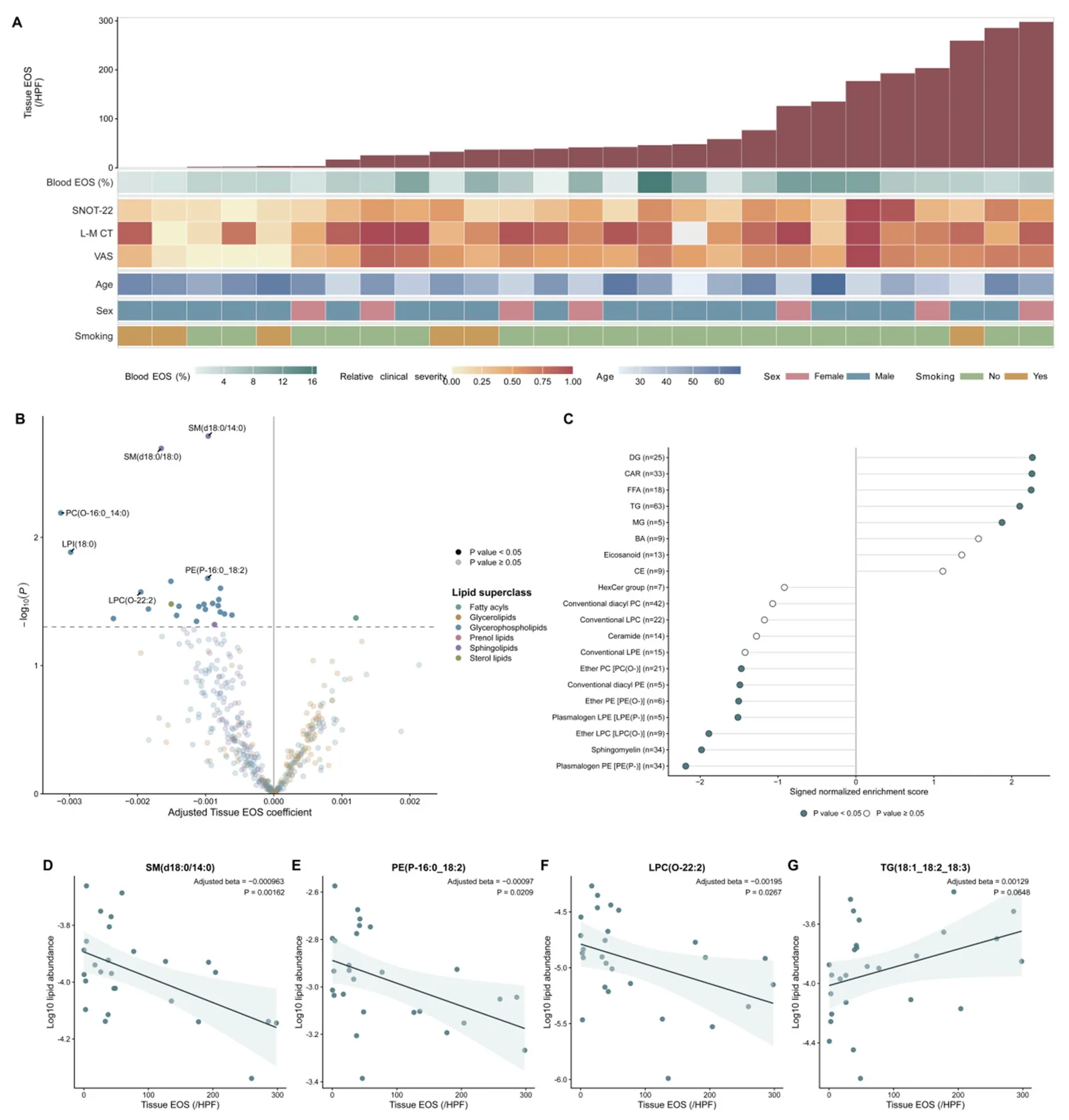
Tissue eosinophilia is associated with coordinated lipid-subclass remodeling within the NP group. (**A**) Clinical and eosinophilic landscape of participants in the NP group ordered by increasing tissue eosinophil counts. Blood eosinophil percentage is shown on its original scale, whereas SNOT-22, overall VAS, and Lund–Mackay CT scores were independently min–max scaled to a 0–1 range for visualization only; statistical analyses used the original values. Age, sex, and smoking status are shown as additional clinical annotations. (**B**) Associations between tissue eosinophil counts and the abundance of 405 individual lipids after adjustment for age, sex, and smoking status; lipids with *p* < 0.05 are highlighted. (**C**) Ranked enrichment of structural lipid subclasses according to their associations with tissue eosinophilia; positive and negative normalized enrichment scores indicate enrichment toward positive and negative lipid-level associations, respectively. Numbers in parentheses indicate the number of retained lipid species in each subclass. (**D**–**G**) Representative associations of tissue eosinophil counts with the sphingomyelin SM(d18:0/14:0), plasmalogen PE(P-16:0_18:2), ether-linked LPC(O-22:2), and TG(18:1_18:2_18:3), respectively. Regression coefficients and *p* values are derived from models adjusted for age, sex, and smoking status. Individual-lipid associations are exploratory; corresponding BH-adjusted *p* values are provided in Supplementary Table S4.

Tissue eosinophil burden varied widely among participants in the NP group and was associated with clinical symptom burden (Figure 4A). Higher tissue eosinophil counts correlated with greater SNOT-22 scores (Spearman ρ = 0.615, *p* = 0.000634) and overall VAS scores (ρ = 0.5419, *p* = 0.0035), whereas the association with Lund–Mackay CT score was weak (ρ = 0.181, *p* = 0.376). Blood eosinophil percentage showed a weaker correlation with tissue eosinophilia (ρ = 0.392, *p* = 0.0430).

In the primary continuous-EOS analysis, 25 of the 405 lipids showed nominal associations with tissue eosinophilia, but none remained significant after BH correction. These individual-lipid associations were therefore considered exploratory. At the individual lipid level, the strongest associations with increasing tissue eosinophilia were largely negative and involved sphingolipid and ether/plasmalogen phospholipid species (Figure 4B). This pattern was exemplified by the sphingomyelin SM(d18:0/14:0), the plasmalogen PE(P-16:0_18:2), and the ether-linked LPC(O-22:2), all of which showed negative associations with tissue eosinophil counts (Figure 4D–F). In contrast, the neutral glycerolipid TG(18:1_18:2_18:3) showed a positive trend with tissue eosinophilia, consistent with the broader TG subclass pattern, although its individual association did not reach *p* < 0.05 (Figure 4G).

Lipid subclass enrichment was negative for plasmalogen PE, sphingomyelin (SM), and ether LPC, and positive for MG, DG, TG, and acylcarnitines, with all these patterns remaining significant after BH correction across the tested subclasses (Figure 4C; Supplementary Table S4). These patterns differed across structurally related subclasses: conventional LPC showed no comparable association, and the negative SM enrichment pattern was not accompanied by corresponding enrichment of Cer or hexosylceramide (HexCer) species.

At the individual lipid level, models using log1p-transformed tissue eosinophil counts and unadjusted Spearman analyses identified 17 and 13 nominally associated lipids respectively. None remained significant after BH correction. All 25 nominal associations from the primary models retained their coefficient directions after log1p transformation, whereas FFA subclass enrichment changed direction and was no longer BH-significant. Leave-one-out analyses preserved the directions of the four representative lipids and the seven evaluated subclass enrichment patterns, despite potentially influential observations in the representative lipid models. The three negative representative lipid associations remained nominally significant in 26–27 of 27 iterations (Supplementary Table S5).

In a complementary comparison using a cutoff of 10 eosinophils/HPF (≥10 vs. <10; *n* = 21 vs. 6), five subclasses showed significant enrichment after BH correction, including negative enrichment of PE(P) and positive enrichment of TG; SM and ether LPC did not remain significant after correction. One TG species, TG(16:0_18:2_20:4), also showed higher relative abundance in the eosinophilic subgroup and remained significant after BH correction across 405 lipids (BH-adjusted *p* = 0.032; Supplementary Table S5).

### 2.5. Clinical Burden Is Associated with Shared and Phenotype-Specific Lipid Remodeling

**Figure 5:**
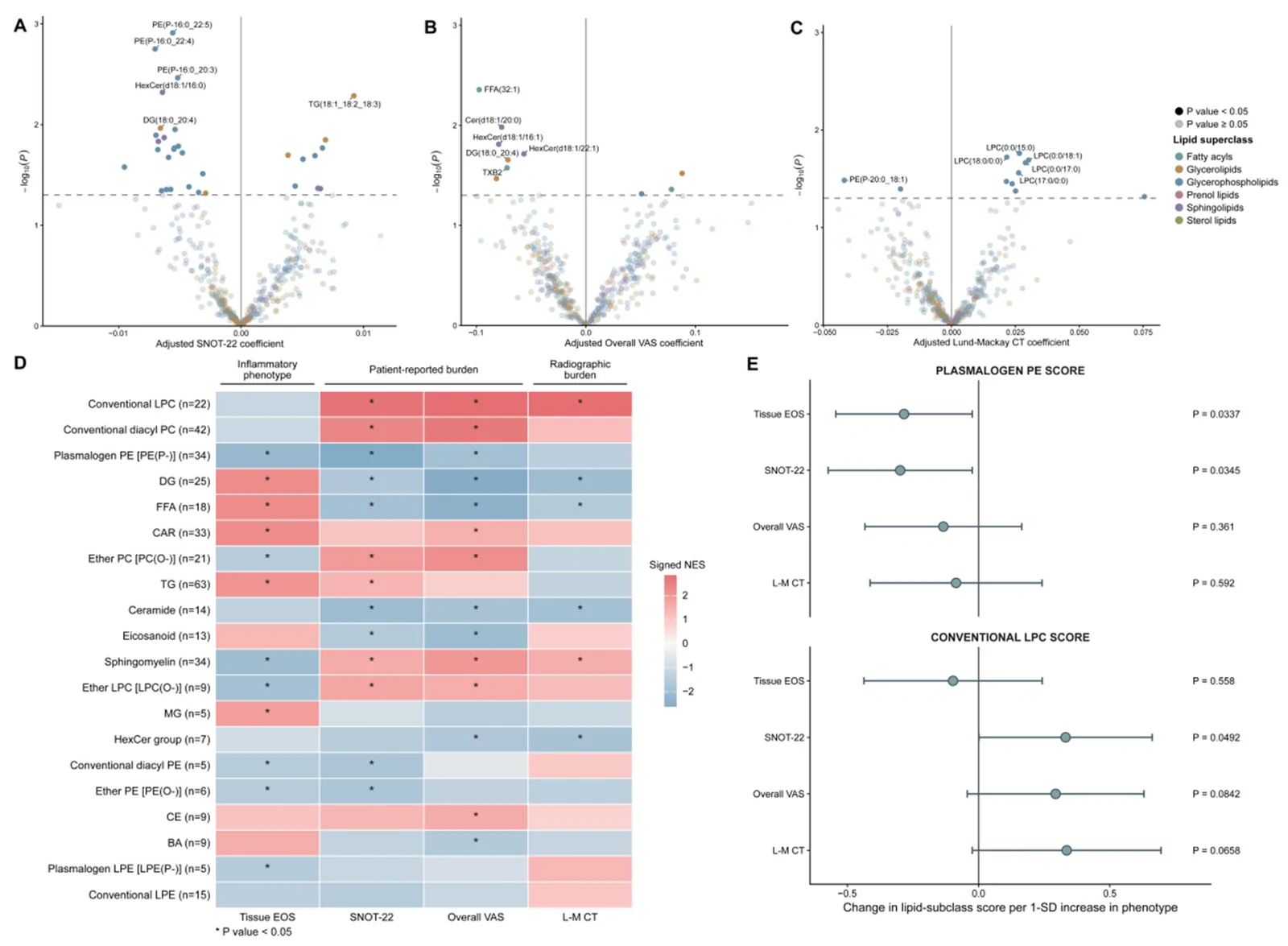
Clinical burden is associated with shared and phenotype-specific lipid remodeling within the NP group. (**A**–**C**) Lipid-wide associations of 405 nasal-fluid lipids with SNOT-22, overall VAS, and Lund–Mackay CT scores, respectively. Regression coefficients were derived from linear models adjusted for age, sex, and smoking status; lipids with *p* < 0.05 are highlighted and colored by lipid superclass. (**D**) Cross-phenotype lipid-subclass landscape comparing ranked enrichment patterns for tissue eosinophil burden, SNOT-22, overall VAS, and Lund–Mackay CT score. Signed normalized enrichment scores (NESs) indicate preferential enrichment of subclass members toward positive or negative phenotype-associated coefficients; asterisks denote *p* < 0.05. Corresponding BH-adjusted *p* values are provided in Supplementary Table S4. Numbers in parentheses indicate the number of retained lipid species in each subclass. (**E**) Patient-level associations of plasmalogen-PE and conventional-LPC subclass scores with the four phenotypes. Subclass scores were calculated for each participant by averaging the standardized abundances of all lipids within the corresponding subclass. Effect estimates represent the change in subclass score per 1-SD increase in each phenotype, with 95% confidence intervals from models adjusted for age, sex, and smoking status. None of the eight focused score–phenotype associations remained significant after BH correction.

At the individual lipid level, patient-reported symptoms and radiographic burden showed different patterns of lipid association. Higher SNOT-22 scores were marked by negative associations among several plasmalogen PE species (Figure 5A), whereas overall VAS showed a broader negative pattern in multiple lipid classes involving sphingolipid, glycerolipid, and inflammatory lipid species (Figure 5B). Lund–Mackay CT score showed a more focused pattern, characterized primarily by positive associations with conventional LPC species together with an inverse association with plasmalogen PE (Figure 5C). Nominal associations were observed for 32 lipids with SNOT-22, 10 with overall VAS, and 11 with Lund–Mackay CT score; none remained significant after BH correction within the corresponding 405-lipid analyses. These individual-lipid patterns were therefore considered exploratory.

Cross-phenotype subclass analysis further clarified the relationship between eosinophilic inflammation and clinical disease burden (Figure 5D). SNOT-22 and overall VAS showed closely aligned subclass profiles, while their overlap with tissue eosinophilia was more selective. The clearest shared feature was negative enrichment of plasmalogen PE across tissue eosinophilia and patient-reported symptom burden. Beyond this common pattern, several subclasses differed markedly across phenotypes. SM shifted from a negative association with tissue eosinophilia to positive associations with symptom burden, whereas DG species showed the opposite pattern. Conventional LPC was similarly distinct, showing little association with tissue eosinophilia but strong positive enrichment with SNOT-22, overall VAS, and Lund–Mackay CT score. Radiographic burden therefore shared the conventional LPC pattern seen with symptom burden but showed little evidence of the eosinophilia-associated plasmalogen PE pattern. Thus, plasmalogen PE represented a shared lipid feature of tissue eosinophilia and patient-reported symptom burden, whereas conventional LPC distinguished clinical and radiographic burden from tissue eosinophilia.

To determine whether these subclass-level patterns were also evident at the patient level, we calculated sample-level scores by averaging the standardized abundances of all lipids within the plasmalogen PE and conventional LPC subclasses (Figure 5E). The plasmalogen PE score decreased with increasing tissue eosinophilia (*p* = 0.0337, FDR = 0.1311) and SNOT-22 burden (*p* = 0.0345, FDR = 0.1311), whereas its associations with overall VAS and Lund–Mackay CT score were weaker. In contrast, the conventional LPC score showed little relationship with tissue eosinophilia but increased with SNOT-22 (*p* = 0.0492, FDR = 0.1311), with similar positive trends for overall VAS and Lund–Mackay CT score. Thus, at the patient level, the plasmalogen PE pattern suggests a potential overlap between eosinophilic inflammation and symptom burden, whereas conventional LPC was preferentially associated with clinical burden and showed little relationship with tissue eosinophilia.

After additional adjustment for atopy, negative enrichment of plasmalogen PE with symptom burden and positive enrichment of conventional LPC with symptom and CT scores remained significant after BH correction. No individual lipid or subclass-score association met BH-adjusted *p* < 0.05 (Supplementary Table S5).

## 3. Discussion

This study identifies both overlapping and divergent patterns of nasal-fluid lipid remodeling across AR and CRSwNP, and examines its associations with local eosinophilic inflammation and clinical burden within the NP group. Differential lipids identified across pairwise group comparisons were organized into four exploratory cross-group modules with distinct abundance patterns, rather than converging on a monotonic CO-AR-NP trajectory. This argues against interpreting AR as an intermediate metabolic state between controls and CRSwNP, while the strong internal coordination within the DG-LPA-related module further indicates that part of the lipid remodeling occurs as an organized program rather than as isolated species-level changes. In the NP group, ranked analysis of associations with tissue eosinophilia showed negative enrichment of PE(P-), SM, and ether-linked LPC and positive enrichment of TG-, DG-, and acylcarnitine subclasses. Reduced PE(P-) was also associated with patient-reported symptom burden, whereas conventional LPC was more closely related to symptomatic and radiographic severity, indicating that local eosinophilic inflammation and clinical burden capture related but non-equivalent dimensions of lipid remodeling. These lipid–burden associations concern variation within the NP group and should not be interpreted as evidence of greater overall symptom burden in CRSwNP than in AR, as the between-group difference in overall VAS was not statistically significant (*p* = 0.372).

At the lipidomic level, the pathophysiological overlap between AR and CRSwNP was selective rather than uniform. Several changes were shared across the two diseases, including increased TG(14:0_18:0_18:2) and reduced free linoleic acid [FFA(18:2)], arachidonic acid [FFA(20:4)], and DL-carnitine relative to controls, although the reductions in FFA and carnitine were generally more pronounced in AR. In contrast, NP showed broader increases in TG and membrane-related lipids, including PE(P-18:0_22:4), TG(16:0_18:2_20:4), ether-linked LPC species, sphingosine, and long-chain acylcarnitines relative to AR. The opposing changes in TG and free PUFA species may reflect redistribution of fatty acids between free and esterified lipid pools. Such dynamic partitioning is biologically plausible because arachidonic acid can be rapidly incorporated into triacylglycerol pools in human inflammatory cells, including eosinophils and mast cells, and subsequently remobilized for phospholipid remodeling and eicosanoid generation [27,28]. However, the present abundance data cannot establish the direction of lipid flux or determine whether the reduced relative abundances of free PUFAs directly contribute to the increased triacylglycerol pool. Previous studies provide partial support for these cross-group patterns. In AR, reduced circulating linoleic acid has been associated with greater symptom burden [14], consistent with the lower nasal-fluid FFA(18:2) observed here, although broader serum lipidomic studies have reported increases in several TG, DG, LPC, and fatty-acid species [16], suggesting that lipid directionality may depend on the molecular species and biological compartment. In CRS, tissue metabolomics has linked eosinophilic CRSwNP to enhanced unsaturated fatty acid oxidation [18], further supporting altered PUFA handling in eosinophilic upper-airway inflammation. Together, these studies support substantial remodeling of fatty acid and complex lipid metabolism in both AR and CRSwNP, while highlighting that the direction of individual lipid changes may vary with inflammatory context and biological compartment.

Module analysis further organized the differential lipids into four exploratory cross-group patterns with differing biochemical compositions. C1, which increased from CO to AR and was highest in NP, was dominated by TGs together with selected ether and plasmalogen phospholipids. Because TGs constitute the major esterified storage form of fatty acids [5,29] and are dynamically linked to FFA availability through esterification and lipolysis [30], this pattern is compatible with progressively greater remodeling of neutral lipid and esterified fatty acid pools, accompanied by comprehensive membrane phospholipid changes in upper-airway inflammation. In contrast, C2 was highest in CO and progressively lower in AR and NP. The module contained DGs and MGs, which serve as intermediates in triacylglycerol synthesis and lipolysis [30,31,32], LPA species regulating cell migration, survival, cytokine production, and tissue remodeling [33], and acylcarnitines involved in mitochondrial trafficking and oxidation of fatty acids [34]. The coordinated decrease in C2 cluster in AR and particularly NP suggests that several related lipid pools involved in glycerolipid turnover, lipid signaling, and fatty acid handling are reduced together. The opposing patterns of C2 and the TG-rich C1 module raise the possibility of a shift in the balance of lipid pools. This may involve lower relative abundance of lipids involved in active turnover and signaling and greater representation of highly esterified neutral storage lipids such as TG. However, the present data measure lipid abundance rather than metabolic flux. Importantly, C2 lipids remained strongly correlated after adjustment for disease group, age, sex, and smoking, indicating that their coordinated variation was not explained solely by the mean differences among CO, AR, and NP. Rather than indicating one discrete pathway, the C2 network may therefore reflect coordinated variation in how fatty acids are distributed among glycerolipid turnover, signaling, and mitochondrial utilization. C3 showed a different pattern, being lowest in AR but highest in NP, characterized by Cer/sphingolipid species together with ether and plasmalogen phospholipids, indicating coordinated changes in remodeling of membrane composition and membrane-associated lipid signaling [5,21,23]. C4, comprising free PUFAs, plasmalogen PE, and oxygenated inflammatory lipids, was also lowest in AR but only partially restored in NP, suggesting altered availability of PUFA substrates and their downstream mediator pools. PUFA species such as arachidonic acid serve as substrates for enzymatic generation of prostaglandins, thromboxanes, leukotrienes, hydroxyeicosatetraenoic acids (HETEs), and other bioactive mediators that regulate both the initiation and resolution of inflammation [8]. Together, these divergent module trajectories argue against a common CO-AR-NP metabolic gradient and instead indicate that AR and CRSwNP remodel distinct lipid systems in different directions. Analysis of all 405 lipids retained several major directional patterns but did not fully reproduce the four-module partition, particularly the original C4 grouping. The modules were therefore interpreted as exploratory patterns within the 147 candidate lipids (Supplementary Table S3).

Within CRSwNP, ranked subclass analysis showed a coordinated pattern of associations with tissue eosinophilia, with negative enrichment of plasmalogen PE, SM, and ether-linked LPC [LPC(O-)] and positive enrichment of TG, DG, and acylcarnitines. This opposing pattern suggests that eosinophilic inflammation is accompanied by redistribution across membrane-associated, neutral lipid, and fatty acid handling pools rather than by a uniform increase or depletion of nasal-fluid lipids. Previous tissue studies provide evidence that long-chain fatty acids accumulate in eosinophilic CRSwNP tissue [18] and can promote eosinophil extracellular DNA trap cell death (EETosis) [35], whereas FFA species in our nasal fluid dataset did not increase uniformly with tissue eosinophilia. This difference may reflect the difference in biological compartment and lipid partitioning, because tissue fatty acid accumulation may occur within cellular or esterified pools without producing a parallel rise in extracellular FFA. The lysophospholipid findings also suggest selective remodeling rather than a uniform change across this lipid class. Lysophosphatidylinositol (LPI), which derives from inositol and signals through GPR55, has recently been reported to increase in eosinophilic CRSwNP tissue and correlates with tissue eosinophilia [36]. Ether-linked LPC belongs to the ether-phospholipid pool and differs from conventional LPC in the chemical linkage of its hydrophobic chain to the glycerol backbone. In our study, ether-linked LPC abundance was inversely associated with tissue eosinophilia, which function in membrane organization, fusion and lipid raft stability [21]. Their opposite associations with eosinophilia therefore suggest that eosinophilic inflammation does not alter all lysophospholipids in a uniform direction but may differentially affect specific membrane-phospholipid remodeling pools. The inverse association of SM with tissue eosinophilia can also be considered in the context of SM-Cer metabolism. Sphingomyelinases hydrolyze SM to generate Cer, whereas SM synthases convert Cer back to SM [37]. Inflammatory cytokines such as TNF-α and IL-1β, growth factors, GPCR signaling and stress signals can activate sphingomyelinases and increase Cer generation [37,38,39]. Fang et al. recently demonstrated Cer, especially Cer(18:1_24:1), accumulating in eosinophilic nasal polyps and involving in eosinophil extracellular-trap formation and release [40]. These findings provide a biological rationale for considering SM–Cer metabolism when interpreting the nasal-fluid SM pattern. However, sphingomyelinase activity was not measured, and a parallel increase in Cer with tissue eosinophilia was not demonstrated in our data. The SM to Cer conversion therefore remains a possible explanation rather than a mechanism established by this study.

Among these changes, the inverse association of PE(P-) with tissue eosinophilia is of particular interest because the plasmalogen vinyl–ether bond is susceptible to oxidative modification [22]. Eosinophil peroxidase-derived brominating species can selectively attack this bond and generate brominated fatty aldehydes in experimental systems [22], providing a plausible mechanism linking eosinophil activation to plasmalogen loss or remodeling. However, reduced PE(P-) in nasal fluid cannot be attributed directly to oxidative consumption because eosinophil peroxidase activity, oxidized plasmalogen products, and lipid flux were not measured. The similar inverse associations of PE(P-) with tissue eosinophilia and patient-reported burden suggest that plasmalogen remodeling may relate to both local eosinophilic inflammation and clinically relevant disease expression.

The different behavior of ether-linked and conventional LPC further highlights the importance of lipid structural context. Ether-linked LPC was inversely associated with tissue eosinophilia, whereas conventional LPC showed little association with tissue eosinophilia but was more strongly associated with patient-reported and radiographic burden. Experimental studies likewise indicate that LPC effects are species- and context-dependent, with selected saturated LPCs suppressing human eosinophil activation [41] while exogenous LPC can promote allergic airway inflammation [42]. Thus, LPCs should not be interpreted as a single functional class. More importantly, the divergence between ether-linked and conventional LPC in our data suggests that they may reflect different dimensions of CRSwNP biology: ether-linked LPC was more closely related to local eosinophilic inflammation, whereas conventional LPC tracked clinical and radiographic disease burden.

The nasal-fluid lipid patterns observed here may reflect reciprocal interactions between local cytokine signaling and lipid metabolism. IL-4 induces LTC_4_ synthase and enhances IgE-dependent cysteinyl leukotriene production in human mast cells [43]. IL-13-induced 15-lipoxygenase-1 contributes to phosphatidylethanolamine oxidation and MUC5AC expression in airway epithelial cells and promotes ERK-dependent CCL26 expression in nasal epithelial cells [44,45]. In turn, PGD_2_ activates group 2 innate lymphoid cells (ILC2s) through CRTH2, and LTE_4_ enhances their responses to PGD_2_ and epithelial cytokines [46,47]. Conversely, PGE_2_ suppresses ILC2 function through EP2/EP4, while IL-13 can downregulate epithelial PGE_2_-related pathways, potentially weakening this inhibitory influence [48,49]. These mechanisms suggest that local lipid remodeling may both result from and modulate inflammatory activity. Our study extended nasal-fluid lipid profiling in AR and CRSwNP and further distinguished how lipid remodeling relates to tissue eosinophilia and disease burden in CRSwNP. In addition, integration of individual lipid species with structural subclasses, cross-group trajectory modules, and correlation networks elucidated the coordinated lipid pattern rather than isolated metabolite changes.

However, this study also has several limitations. The single-center cohort was relatively modest in size, particularly for CRSwNP-specific analyses, which may have limited power after multiple-testing correction and the precision of effect estimates. The small non-eosinophilic subgroup (*n* = 6) further limits the precision of threshold-based comparisons. Although some between-group differences and subclass enrichments survived their respective BH corrections, individual-lipid and focused score associations within CRSwNP did not. Previous sinonasal surgery and disease duration were not included as covariates in the within-CRSwNP models, and residual confounding by these factors cannot be excluded. Targeted acquisition and unequal lipid-class coverage also limit extrapolation of the subclass-level findings beyond the measured lipid set. Validation in larger multicenter cohorts and targeted LC–MS/MS confirmation with isotope-labeled internal standards would strengthen these findings. The absence of a CRSsNP group limits assessment of whether the observed lipidomic alterations are specific to CRSwNP. Moreover, residual effects of diagnostic heterogeneity within the surgical control group cannot be excluded. Although all AR patients underwent diagnostic allergen testing, the specific sensitizing allergens and seasonal versus perennial AR classification were not recorded, limiting our ability to assess lipidomic differences across these AR subgroups. AR sampling during the local spring allergy season also limits generalizability to other seasonal contexts. Also, nasal fluid composition does not fully recapitulate the underlying tissue microenvironment [25], and bulk nasal fluid profiling cannot assign individual lipids to specific cellular sources. Therefore, paired tissue–nasal fluid lipidomics, spatial metabolomics, or lipid profiling of isolated cell populations could help resolve the cellular and tissue sources of the measured lipids. Finally, because the present measurements reflect lipid abundance rather than metabolic flux, they cannot determine whether observed changes arise from altered synthesis, degradation, secretion, or uptake. Stable-isotope tracing and targeted mechanistic studies could be needed to test the metabolic relationships suggested by the present findings.

In conclusion, nasal fluid lipidomics revealed both shared and disease-specific lipid remodeling across AR and CRSwNP, with differential lipids organizing into coordinated modules rather than a uniform CO-AR-NP gradient. Within CRSwNP, tissue eosinophilia was associated with a distinct pattern characterized by negative enrichment of plasmalogen PE, SM, and ether-linked LPC and positive enrichment of glycerolipid and acylcarnitine subclasses. Clinical burden showed partially overlapping lipid associations, with plasmalogen PE linked to both eosinophilic inflammation and symptom burden, whereas conventional LPC was more closely related to patient-reported symptom and radiographic severity. These findings highlight the potential value of nasal fluid profiling for characterizing lipid remodeling in upper-airway inflammation and provide a basis for future targeted validation and mechanistic studies.

## 4. Materials and Methods

### 4.1. Study Design and Participants

This cross-sectional observational lipidomics study consecutively enrolled 73 participants from the Department of Otorhinolaryngology of Peking Union Medical College Hospital (PUMCH) between January and April 2021. Eligible participants were 18–80 years of age and were classified into three independent groups: controls (CO; *n* = 22), allergic rhinitis (AR; *n* = 23), and CRSwNP (NP; *n* = 28).

Patients in the NP group fulfilled the diagnostic criteria for CRSwNP from the European Position Paper on Rhinosinusitis and Nasal Polyps 2020 (EPOS 2020) [50], including chronic sinonasal symptoms persisting for at least 12 weeks together with objective evidence of sinonasal inflammation, with the presence of nasal polyps confirmed by nasal endoscopy. AR was diagnosed according to international consensus criteria [51], based on a compatible history of allergen-related nasal symptoms, including sneezing, rhinorrhea, nasal itching, and/or nasal obstruction, together with evidence of sensitization to relevant aeroallergens by skin-prick testing and/or serum allergen-specific IgE. Participants assigned to the isolated AR group had no clinical, endoscopic, or radiological evidence of chronic rhinosinusitis or nasal polyps. Classification as seasonal or perennial AR and detailed allergen-specific sensitization profiles were not systematically recorded in the study dataset.

The CO group consisted of participants undergoing endoscopic endonasal surgery for noninflammatory conditions, including isolated nasal septal deviation, paranasal sinus cysts, or nonfunctioning pituitary adenomas. Controls had no history or clinical evidence of allergic rhinitis, chronic rhinosinusitis, nasal polyps or asthma. Preoperative nasal endoscopy and/or computed tomography (CT) showed no evidence of sinonasal inflammatory disease, and hematoxylin and eosin (H&E) staining of sinonasal tissue specimens did not reveal significant inflammatory changes.

Exclusion criteria for all participants were systemic or intranasal corticosteroid use within 1 month before enrollment, oral antihistamine use within 2 weeks before enrollment, unilateral sinusitis, fungal rhinosinusitis, immunodeficiency disorders, or other conditions considered likely to confound the inflammatory or metabolic profile. The study was approved by the Ethics Committee of PUMCH. All participants provided written informed consent before enrollment and sample collection.

### 4.2. Clinical Phenotyping

Clinical and demographic variables recorded at enrollment included age, sex, smoking history, disease duration, comorbid allergic rhinitis and asthma, previous sinonasal surgery, and atopic status. Atopy was defined by the presence of sensitization to one or more aeroallergens based on serum allergen-specific IgE testing. Laboratory variables included the peripheral white blood cell count (×10^9^/L), blood eosinophil percentage, absolute blood eosinophil count (×10^9^/L), and total serum immunoglobulin E (IgE; kU/L). Clinical disease burden was assessed using an overall sinonasal symptom visual analog scale (VAS; 0–10), individual symptom-specific VAS scores (0–10) [52], the 22-item Sinonasal Outcome Test (SNOT-22) [53], and the Lund–Mackay computed tomography (CT) score [54]. The overall VAS reflected each participant’s global perceived sinonasal symptom burden (annotated as overall VAS), whereas the symptom-specific VAS scores (0–10) assessed nasal obstruction, rhinorrhea, smell reduction, and facial pain/pressure separately. Higher VAS scores indicated greater symptom burden. Health-related quality of life was assessed using the Chinese version of the SNOT-22 [55], in which each of the 22 items is scored from 0 to 5, yielding a total score of 0–110; higher scores indicate greater symptom and quality-of-life burden. Radiological disease extent was evaluated using the Lund–Mackay CT scoring system, with total scores ranging from 0 to 24 and higher scores indicating greater radiographic disease burden. Two otorhinolaryngologists independently assigned Lund–Mackay scores from the CT images while blinded to all other participant information. Discrepancies were adjudicated by a third, more senior physician. In the AR group, clinical assessments and nasal fluid collection were performed at enrollment. In the NP and CO groups, clinical assessments and investigations were performed at hospital admission, and nasal fluid was collected before surgery. Missing observations were retained as missing and were not imputed for clinical association analyses.

### 4.3. Nasal Fluid Collection

Nasal fluid was collected bilaterally using a previously described sponge-based method [56]. No nasal irrigation or cleansing was performed, and no topical anesthetic or vasoconstrictor was applied before collection in any group. Trimmed Merocel sinus sponges (Medtronic Xomed, Jacksonville, FL, USA) were placed in the middle meatus of each side, parallel to the sagittal plane, and left in place for 5 min. Following collection, 1 mL of 0.9% saline was added to the sponge to elute the absorbed secretions. The sponges were maintained at 4 °C for 2–24 h and subsequently transferred to 5 mL syringes. Nasal secretions were recovered by centrifugation at 1500× *g* for 10–15 min at 4 °C, transferred into sterile microcentrifuge tubes, aliquoted and stored at −80 °C until lipidomic analysis. All nasal-fluid samples from participants with AR were collected during the local spring allergy season.

### 4.4. Tissue Eosinophil Quantification

Sinonasal tissue specimens were obtained intraoperatively from participants in the NP and control groups; no tissue specimens were collected from participants with isolated AR. All tissues were fixed in 10% neutral-buffered formalin, paraffin-embedded, sectioned, and stained with hematoxylin and eosin (H&E). In the control group, histological examination was used to exclude significant sinonasal inflammatory changes. In the NP group, tissue eosinophil burden was quantified on H&E-stained sections. Two experienced pathologists, blinded to the participants’ clinical information, independently evaluated five randomly selected high-power fields (HPFs; ×400 magnification) per specimen. Eosinophils were counted in each field, and the mean eosinophil count across the five fields was calculated for each observer. The final tissue eosinophil value was defined as the average of the two observers’ mean counts and expressed as eosinophils per HPF. Tissue eosinophil measurements were available for 27 of 28 participants in the NP group and were used as a continuous variable in subsequent eosinophil-associated analyses.

### 4.5. Lipid Extraction

Frozen nasal-fluid samples were thawed and vortex-mixed for 10 s. A 100 μL aliquot of each sample was transferred to a 2 mL centrifuge tube and mixed with 1 mL of an internal-standard-containing extraction solution, followed by vortexing for 3 min. Subsequently, 200 μL of ultrapure water was added, and the mixture was vortexed for an additional 3 min and incubated on ice for 15 min. Samples were centrifuged at 12,000 rpm for 10 min at 4 °C, after which 500 μL of the supernatant was transferred to a new 1.5 mL centrifuge tube and concentrated to dryness. The dried extract was reconstituted in 200 μL of isopropanol/acetonitrile (1:1, *v*/*v*), vortexed for 5 min, and centrifuged at 12,000 rpm for 3 min at 4 °C. Finally, 100 μL of the supernatant was transferred to an autosampler vial for LC–MS/MS analysis.

### 4.6. UPLC–ESI–MS/MS Analysis

Lipid extracts were analyzed using an ExionLC AD ultraperformance liquid chromatography system coupled to a SCIEX QTRAP triple-quadrupole linear ion-trap mass spectrometer operated in both positive- and negative-ionization modes. Chromatographic separation was performed on a Thermo Accucore C30 column (2.1 × 100 mm, 2.6 μm, Thermo Fisher Scientific, Waltham, MA, USA) maintained at 45 °C. Mobile phase A consisted of acetonitrile/water (60:40, *v*/*v*) containing 0.1% formic acid and 10 mmol/L ammonium formate, and mobile phase B consisted of acetonitrile/isopropanol (10:90, *v*/*v*) containing 0.1% formic acid and 10 mmol/L ammonium formate. The gradient program was as follows: 80:20 (A:B) at 0 min, 70:30 at 2.0 min, 40:60 at 4.0 min, 15:85 at 9.0 min, 10:90 at 14.0 min, 5:95 at 15.5–17.3 min, and 80:20 at 17.5–20.0 min. The flow rate was 0.35 mL/min, and the injection volume was 2 μL.

Linear ion trap (LIT) and triple-quadrupole (QQQ) scans were acquired on the QTRAP system. The Turbo IonSpray source was operated at 500 °C with ion-spray voltages of +5500 V and −4500 V in positive- and negative-ion modes, respectively. Ion-source gas 1, ion-source gas 2, and curtain gas were set to 45, 55, and 35 psi, respectively. The collision gas setting was medium. Instrument tuning and mass calibration were performed using polypropylene glycol solutions at 10 and 100 μmol/L in triple-quadrupole and linear ion-trap modes, respectively. QQQ scans were acquired in multiple-reaction-monitoring (MRM) mode with nitrogen as the collision gas at 5 psi. Declustering potentials and collision energies were optimized for individual MRM transitions. Specific sets of MRM transitions were monitored during acquisition periods according to the retention times of the eluting lipids. Data acquisition was controlled using Analyst 1.6.3 software (SCIEX). Available MRM parameters are provided in Supplementary Table S1. Chromatographic peaks were integrated using MultiQuant, and integrations were reviewed across samples based on retention times and peak shapes. The resulting peak areas were used as measures of relative lipid abundance.

Lipid extraction, LC–MS/MS acquisition, lipid annotation, lipid class/subclass assignment, and chromatographic peak integration were performed by MetWare Biotechnology (Wuhan, China). The resulting peak-area data were subsequently subjected to total-peak-area normalization, quality-control filtering, and statistical analyses by the authors, as described below.

### 4.7. Quality Control and Preprocessing

A pooled quality-control (QC) sample was prepared by combining aliquots of individual sample extracts and was repeatedly analyzed throughout the LC–MS/MS sequence, with one pooled-QC injection inserted after every 10 analytical samples. Ten pooled-QC injections (QC01–QC10) were obtained across the analytical run and were used to assess analytical reproducibility and signal stability. Pairwise Spearman correlation coefficients among QC injections were calculated separately for positive- and negative-ion modes, and lipid-specific coefficients of variation (CVs) were calculated across the ten pooled-QC injections using untransformed, total-peak-area-normalized relative abundances.

A total of 465 quantified lipid species were subjected to quality-control filtering. Lipids were retained for downstream analysis if they were detected in at least 80% of biological samples and had a pooled-QC CV ≤ 30%. The supplied matrix contained no missing or zero-valued entries, so no imputation or zero replacement was performed. Sixty lipids with pooled-QC CV > 30% were excluded, leaving 405 lipid species for downstream analyses (Supplementary Table S1).

Sample processing was volume-standardized using a fixed 1 mL volume of 0.9% saline for elution and a fixed 100-μL aliquot of the recovered eluate for lipid extraction. For each sample, the integrated peak area of each lipid was divided by the sum of the peak areas of all 465 quantified lipid species in that sample to obtain total-peak-area-normalized relative abundances. After QC filtering, 405 lipid species were retained and their relative abundances were log10-transformed for downstream statistical analyses. As the normalized values were between 0 and 1, the resulting log10-transformed values were negative. No normalization to total protein or independently measured total lipid content was performed. Residual variation in sample collection, dilution, and lipid-specific extraction recovery cannot be fully excluded. Sample-level distributions of log10-transformed lipid abundances were summarized descriptively. For multivariate and functional-program analyses, log10-transformed lipid abundances were mean-centered and scaled to unit variance.

### 4.8. Lipid Annotation and Classification

Lipid species were annotated by MetWare Biotechnology (Wuhan, China) using its proprietary in-house MetWare Database (MWDB), based on reference retention times and characteristic precursor/product-ion transitions [57,58,59]. Molecular formulae were not independently established by accurate-mass measurements in this study. The reference information used to establish the targeted methods included available authentic standards, public spectral resources, and established lipid fragmentation rules. For the reported PE(P−), PE(O−), and LPC(O−) species without corresponding authentic standards, MRM methods were established using public spectral references and fragmentation rules, including those from LipidBlast [60]. Not all lipid assignments were confirmed using authentic standards. For downstream analyses, the provider-reported lipid annotations and class/subclass assignments were organized with reference to established lipid-classification and nomenclature frameworks, including LIPID MAPS [61,62]. The retained lipid species were assigned to six major lipid categories: fatty acyls, glycerolipids, glycerophospholipids, sphingolipids, sterol lipids, and prenol lipids. Conventional lysophosphatidylcholines (LPC), ether-linked LPC [LPC(O-)], plasmalogen phosphatidylethanolamines [PE(P-)], ether-linked phosphatidylethanolamines [PE(O-)] and conventional diacyl PE species were treated as separate structural subclasses according to the provider-reported annotations. Detailed lipid annotations, subclass and program assignments, quality-control metrics, and processed abundances are provided in Supplementary Table S1.

To summarize coordinated lipid changes at a biologically interpretable level, lipid species were additionally assigned to nine study-defined structural–functional programs: lysophospholipid remodeling, fatty-acid oxidation/acylcarnitine, ether/plasmalogen phospholipids, free fatty acid (FFA)/PUFA, sterol/bile acid, eicosanoid/oxidized lipid, neutral-lipid storage, sphingolipid remodeling, and diacyl membrane phospholipids. Program assignment was based on lipid class, molecular structure, and established biochemical context and was guided by established lipid-classification and ontology frameworks [63]. These programs were used as descriptive summaries of coordinated lipid patterns and were not interpreted as direct measurements of metabolic pathway activity or flux.

For each lipid, log10-transformed relative abundances were centered and scaled to unit variance across all 73 participants. Each participant’s program score was the unweighted mean Z-score of the retained lipids assigned to that program. Heatmap values were group means estimated from linear models adjusted for age, sex, and smoking, with covariates held at their cohort means. These values were displayed without further standardization, using fixed row and column orders without clustering. Pairwise group differences in program scores were evaluated using moderated *t*-tests in limma (version 3.64.3) [64], with BH correction across the nine programs separately within each comparison.

### 4.9. Multivariate Analysis

PCA was performed on the same log10-transformed, lipid-wise autoscaled abundance matrix. Binary orthogonal partial least squares–discriminant analysis (OPLS-DA) [65] was performed on the log10-transformed, lipid-wise autoscaled abundance matrix using the opls function in the R package ropls (version 1.40.0) [66]. Each model contained one predictive component and was evaluated using sevenfold cross-validation and 200 label permutations. The AR-versus-CO, NP-versus-AR, and NP-versus-CO models contained two, four, and three orthogonal components, respectively. Model performance was summarized using cumulative R2X, R2Y, and Q2 values, together with permutation-derived *p* values for R2Y and Q2 (Supplementary Figure S3 and Supplementary Table S2).

### 4.10. Differential Lipid Analysis

Lipid-wise differences among clinical groups were assessed using empirical-Bayes moderated linear models implemented in the limma package (version 3.64.3) in R. For each lipid species, log10-transformed abundance was modeled as a function of clinical group with adjustment for age, sex, and smoking status. Pairwise contrasts were specified for NP versus CO, AR versus CO, and NP versus AR. For each pairwise contrast, the covariate-adjusted coefficient on the log10 scale was multiplied by log2(10) to obtain the adjusted log2 fold change. Standard errors were converted using the same factor, and 95% Wald confidence intervals were calculated as log2FC ± 1.96 × SElog2.

A two-sided *p* value < 0.05 was used as the threshold for exploratory visualization of lipid patterns. Benjamini–Hochberg (BH) correction was applied across the 405 lipid species separately within each pairwise comparison, with BH-adjusted *p* < 0.05 identifying findings supported after correction for multiple testing. A curated panel of eight representative lipids spanning major lipid classes and biologically interpretable patterns was additionally used to summarize pairwise effect estimates and 95% confidence intervals across the three comparisons (Supplementary Table S2).

### 4.11. Lipid Trajectory Clustering and Network Analysis

The module input comprised 147 lipids reaching *p* < 0.05 in at least one age-, sex-, and smoking-adjusted pairwise comparison among CO, AR, and NP. Covariate-adjusted CO, AR, and NP effects were standardized within each lipid across the three groups and subjected to K-means clustering. Candidate K values from 2 to 9 were assessed using total within-cluster sum of squares, mean silhouette width calculated with the cluster package (version 2.1.8.1), cluster-size structure, and co-assignment stability across 200 repetitions using 80% lipid subsampling. K = 4 was retained as a parsimonious and biologically interpretable solution among the 147 candidate lipids, with near maximal silhouette performance (0.6106; maximum 0.6122 at K = 6) and high subsampling stability (0.9795; maximum 0.9889 at K = 2) (Supplementary Figure S4 and Supplementary Table S3). To assess dependence on significance-based lipid selection, the same clustering procedure was repeated using all 405 retained lipids without *p*-value filtering, and K = 4 assignments were compared for the 147 shared lipids (Supplementary Table S3).

For network analysis, residuals were obtained for each lipid from linear models including group, age, sex, and smoking status. Pairwise Spearman correlations were calculated among residuals, and edges were retained for |ρ| ≥ 0.80 and *p* < 0.05. Network construction and analysis were performed using igraph (version 2.3.3) [67]. Edge weights were defined as |ρ|, and weighted Newman–Girvan modularity was calculated for the fixed K-means partition. The within-module edge proportion and modularity were evaluated using 10,000 size-preserving permutations of the module labels. The |ρ| ≥ 0.80 cutoff was used to focus on strong residual associations. Threshold sensitivity was assessed using cutoffs of 0.70 and 0.85 while retaining the same 147 lipids, module assignments, and *p*-value criterion (Supplementary Table S3). Cluster-specific Kyoto Encyclopedia of Genes and Genomes (KEGG) over-representation analysis was performed using the KEGG-annotated lipids within the analyzed lipid set as the background universe. KEGG results were interpreted as putative biological associations based on lipid annotations, with caution regarding sparse or ambiguous mappings, rather than as evidence of pathway activation or metabolic flux (Supplementary Table S3).

### 4.12. Lipid Phenotype Association Analysis Within the NP Group

Within the NP group, associations between lipid abundance and tissue eosinophilia or clinical disease burden were evaluated using covariate-adjusted linear regression models. For tissue eosinophilia, log10-transformed lipid abundance was modeled as a function of the continuous tissue eosinophil count (/HPF), with adjustment for age, sex, and smoking status. Twenty-seven participants had complete tissue-eosinophil and covariate data. Figure 4B displays the unstandardized regression coefficient for the tissue eosinophil count, representing the adjusted difference in log10 relative lipid abundance per additional eosinophil/HPF. Clinical association models separately evaluated SNOT-22, overall VAS, and Lund–Mackay CT scores using the same covariate adjustment and phenotype-specific complete cases. For each phenotype, regression coefficients, standard errors, 95% confidence intervals, and *p* values were obtained for all 405 lipid species (Supplementary Table S4). Benjamini–Hochberg correction was applied separately across the 405 lipid-level tests for each phenotype, and the corresponding adjusted *p* values are also reported in Supplementary Table S4.

Four representative lipids were selected using a rule-based and curated procedure that prioritized complementary lipid subclass coverage, adjusted tissue-eosinophil associations, cross-analysis directional consistency, and robustness in influence and leave-one-out analyses. These representative lipids were used to illustrate coordinated subclass-level patterns and were not interpreted as independently validated markers.

### 4.13. Lipid-Subclass Ranked Enrichment

For each phenotype, all 405 lipid species were ranked according to the regression t statistic (β/SE) derived from the corresponding covariate-adjusted lipid-level models. Structural lipid subclasses containing 5–200 measured lipid species were evaluated using competitive ranked enrichment with the fgseaSimple function implemented in the fgsea package (version 1.34.2) [68], following the general framework of gene-set enrichment analysis. Enrichment significance was estimated using 100,000 permutations and signed normalized enrichment scores (NESs) and *p* values were reported. BH-adjusted *p* values were calculated across the tested structural subclasses separately for each phenotype. A positive NES indicated preferential enrichment of subclass members toward positive phenotype-associated coefficients, whereas a negative NES indicated enrichment toward negative coefficients. Subclass enrichment was interpreted as coordinated directional lipid remodeling rather than as evidence of metabolic flux or a shared cellular origin (Supplementary Table S4).

### 4.14. Focused Lipid-Subclass Score Analysis

Focused sample-level scores were calculated for plasmalogen PE [PE(P-); 34 lipids] and conventional LPC (22 lipids). Within the NP group, each constituent lipid was standardized across participants, and the subclass score was calculated as the mean Z score across all subclass members. Each subclass score was then modeled separately against the tissue eosinophil count, SNOT-22, overall VAS, and Lund–Mackay CT score, with each phenotype standardized so that model coefficients represented the change in subclass score per 1-SD increase in the phenotype. Models were adjusted for age, sex, and smoking status, and effect estimates, 95% confidence intervals, and *p* values were reported. Benjamini–Hochberg correction was applied across the eight focused score–phenotype tests. These score-based analyses were used as complementary summaries of mean subclass abundance and were interpreted separately from the ranked-enrichment analysis (Supplementary Tables S1 and S4).

### 4.15. Sensitivity Analyses

Between-group analyses were repeated after excluding participants with asthma. Within the NP group, models with and without atopy were compared in identical complete cases, retaining the original lipid set, score definitions, and BH correction procedures (Supplementary Table S5).

Sensitivity analyses for tissue-eosinophil associations included alternative log1p transformation of tissue eosinophil counts, unadjusted Spearman analyses, influence diagnostics, and leave-one-participant-out analyses to evaluate the stability of lipid- and subclass-level associations. Concordance of lipid-level effect direction and ranked subclass patterns was additionally examined across sensitivity analyses (Supplementary Table S5).

Threshold-based sensitivity analyses used ≥10 versus <10 eosinophils/HPF, with binary endotype replacing continuous tissue EOS in otherwise identical adjusted models and ranked subclass analyses (Supplementary Table S5).

### 4.16. Statistical Analysis

Continuous variables were summarized as median [interquartile range], and categorical variables as n/N (%). Three-group continuous comparisons were performed using Kruskal–Wallis tests, pairwise continuous comparisons using Wilcoxon rank-sum tests, and categorical comparisons using Fisher’s exact tests, as appropriate. Unless otherwise specified, all tests were two-sided. Nominal and BH-adjusted *p* values were reported separately, with multiple-testing correction applied within the analysis-specific test families described above. Analyses were performed in R version 4.5.0.

## Figures and Tables

**Table 1:** Demographic and clinical characteristics of the study population. Data are presented as median [interquartile range] for continuous variables and n/N (%) for categorical variables. For variables available across CO, AR, and NP, continuous variables were compared using the Kruskal–Wallis test and categorical variables using Fisher’s exact test. For variables available only in AR and NP, including disease duration, continuous variables were compared using the Wilcoxon rank-sum test and categorical variables using Fisher’s exact test. Denominators reflect the number of participants with available data for each variable. “—“ indicates that the variable was not applicable or not collected in the corresponding group. Overall VAS denotes the separately recorded overall sinonasal symptom visual analog scale (0–10) and is distinct from the individual VAS scores for nasal obstruction, rhinorrhea, smell reduction, and facial pain/pressure. Atopy was defined as sensitization to at least one aeroallergen by serum allergen-specific IgE testing.

Variable	Overall	CO	AR	NP	*p* Value
Age, years	35.0 [27.0, 53.0]	32.0 [27.0, 44.5]	31.0 [27.0, 40.0]	49.0 [33.8, 55.2]	0.004
Male sex, n/N (%)	51/73 (69.9%)	19/22 (86.4%)	12/23 (52.2%)	20/28 (71.4%)	0.040
Smoking history, n/N (%)	11/73 (15.1%)	0/22 (0.0%)	5/23 (21.7%)	6/28 (21.4%)	0.037
Disease duration, years	6.0 [3.0, 10.0]	—	7.0 [3.0, 15.0]	5.5 [2.8, 7.8]	0.294
Comorbid allergic rhinitis, n/N (%)	12/28 (42.9%)	—	—	12/28 (42.9%)	
Comorbid asthma, n/N (%)	9/51 (17.6%)	—	1/23 (4.3%)	8/28 (28.6%)	0.031
Previous surgery, n/N (%)	4/28 (14.3%)	—	—	4/28 (14.3%)	
Atopy, n/N (%)	30/47 (63.8%)	—	23/23 (100.0%)	7/24 (29.2%)	<0.001
White blood cell count	6.8 [6.0, 7.8]	6.2 [5.2, 7.8]	6.9 [6.3, 8.1]	6.8 [6.0, 7.8]	0.486
Blood eosinophils, %	3.9 [1.9, 5.4]	2.2 [1.2, 2.9]	3.8 [2.5, 5.0]	5.0 [3.1, 8.6]	0.006
Blood eosinophil count, ×10^9^/L	0.3 [0.1, 0.5]	0.1 [0.1, 0.3]	0.2 [0.2, 0.3]	0.4 [0.2, 0.5]	0.022
Total IgE	94.7 [41.7, 256.8]	—	94.7 [62.6, 224.5]	86.5 [32.2, 324.2]	0.603
Nasal blockage score	8.0 [6.0, 9.0]	—	8.0 [4.0, 8.5]	8.0 [6.5, 9.0]	0.385
Rhinorrhea score	7.0 [6.0, 8.0]	—	7.0 [5.0, 8.0]	7.0 [6.0, 8.0]	1.000
Smell reduction score	6.0 [0.0, 10.0]	—	0.0 [0.0, 1.5]	10.0 [8.0, 10.0]	<0.001
Facial pain/pressure score	0.0 [0.0, 0.0]	—	0.0 [0.0, 0.0]	0.0 [0.0, 1.5]	0.026
Overall VAS score	6.5 [6.0, 7.0]	—	6.0 [5.5, 7.0]	7.0 [6.0, 7.5]	0.372
SNOT-22	37.0 [21.0, 49.0]	—	—	37.0 [21.0, 49.0]	
Lund–Mackay score	20.0 [16.0, 22.0]	—	—	20.0 [16.0, 22.0]	

## Data Availability

The raw data supporting the findings of this study have been deposited in Science Data Bank (ScienceDB). Processed lipidomic data and analysis results supporting the principal findings are provided in the Supplementary Materials.

## Supplementary Materials

The following supporting information can be downloaded at https://www.mdpi.com/article/10.3390/ijms27219715/s1.

## Abbreviations

The following abbreviations are used in this manuscript:

AR	Allergic rhinitis
Cer	Ceramide
CI	Confidence interval
CO	Control
CRS	Chronic rhinosinusitis
CRSwNP	Chronic rhinosinusitis with nasal polyps
CT	Computed tomography
CV	Coefficient of variation
DG	Diacylglycerol
FFA	Free fatty acid
H&E	Hematoxylin and eosin
HPF	High-power field
IgE	Immunoglobulin E
IL	Interleukin
KEGG	Kyoto Encyclopedia of Genes and Genomes
LC–MS/MS	Liquid chromatography–tandem mass spectrometry
LPA	Lysophosphatidic acid
LPC	Lysophosphatidylcholine
LPC(O−)	Ether-linked lysophosphatidylcholine
LPE	Lysophosphatidylethanolamine
LPI	Lysophosphatidylinositol
MG	Monoacylglycerol
NES	Normalized enrichment score
NP	CRSwNP study group
OPLS-DA	Orthogonal partial least squares–discriminant analysis
PC	Phosphatidylcholine
PE	Phosphatidylethanolamine
PE(O−)	Ether-linked phosphatidylethanolamine
PE(P−)	Plasmalogen phosphatidylethanolamine
PUFA	Polyunsaturated fatty acid
QC	Quality control
SD	Standard deviation
SE	Standard error
SM	Sphingomyelin
SNOT-22	22-item Sino-Nasal Outcome Test
TG	Triacylglycerol
TXB2	Thromboxane B2
UPLC–ESI–MS/MS	Ultra-performance liquid chromatography–electrospray ionization–tandem mass spectrometry
VAS	Visual analog scale
