# Supplementary material for: Widely Targeted Lipidomics of Nasal Fluid Across Upper-Airway Inflammation: Phenotype-Associated Remodeling in Allergic Rhinitis and Chronic Rhinosinusitis with Nasal Polyps
Source: Int J Mol Sci. 2026 Oct 29;27(21):9715. doi: 10.3390/ijms27219715 (PMC13763025; doi:10.3390/ijms27219715)
Supplement: Supplementary file 1 [file ijms-27-09715-s001.zip › Supplementary_Figure S2_program_pairwise_CI.pdf]

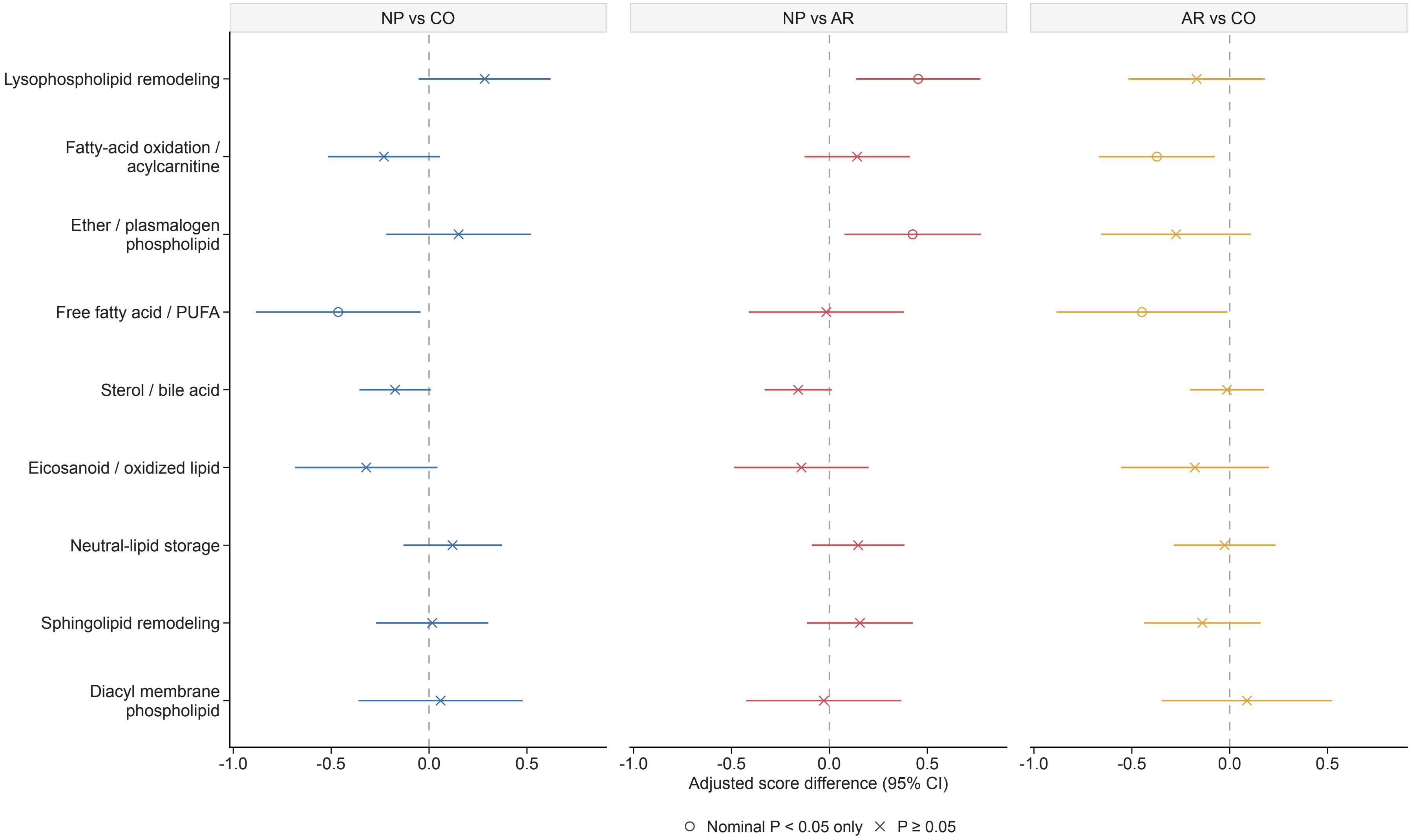

Supplementary Figure S2. Adjusted between-group differences in structural-functional lipid-program scores.

Points show between-group differences adjusted for age, sex, and smoking status; horizontal bars indicate approximate 95% confidence intervals (estimate ±1.96 SE). Positive values indicate higher scores in the first-named group. Circles indicate nominal $P < 0.05$ and crosses indicate $P \geq 0.05$. P values are from moderated t-tests, with BH correction across nine programs separately within each comparison. No pairwise comparison met BH $q < 0.05$.
