# Supplementary material for: Widely Targeted Lipidomics of Nasal Fluid Across Upper-Airway Inflammation: Phenotype-Associated Remodeling in Allergic Rhinitis and Chronic Rhinosinusitis with Nasal Polyps
Source: Int J Mol Sci. 2026 Oct 29;27(21):9715. doi: 10.3390/ijms27219715 (PMC13763025; doi:10.3390/ijms27219715)
Supplement: Supplementary file 1 [file ijms-27-09715-s001.zip › Supplementary_Figure_S3_OPLSDA_validation.pdf]

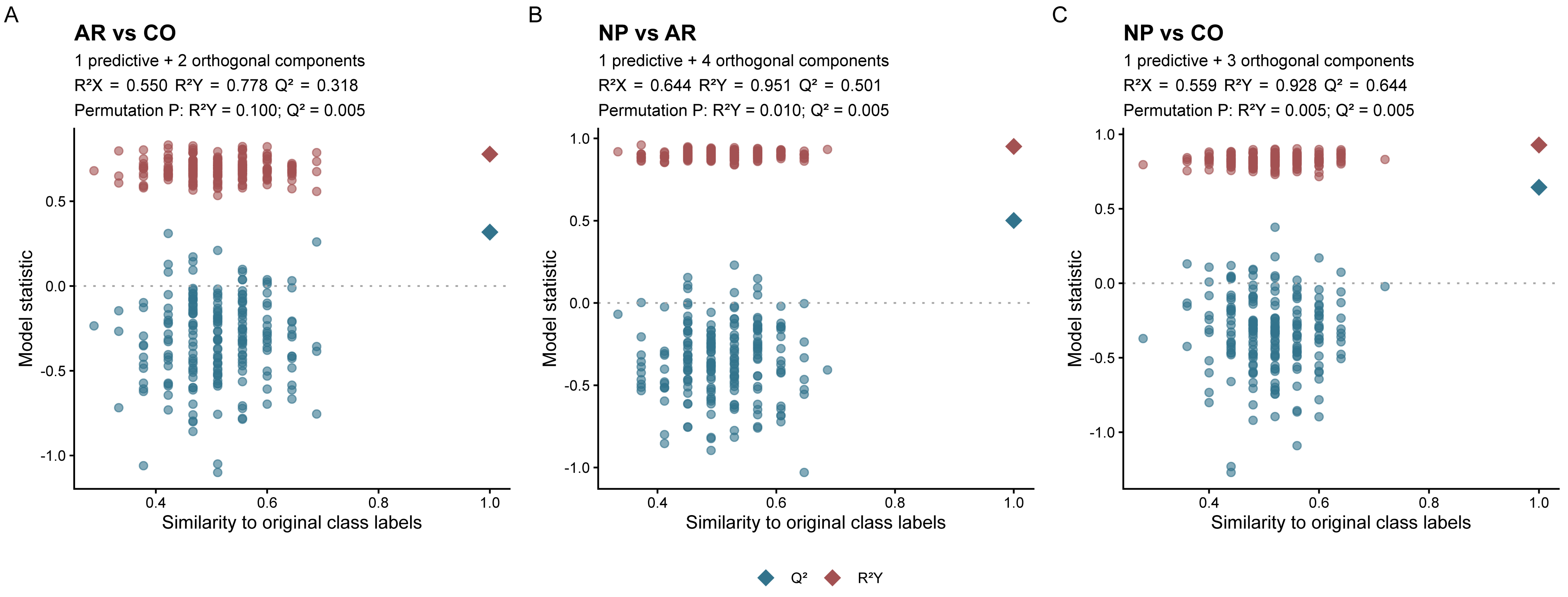

Supplementary Figure S3. Permutation validation of pairwise OPLA-DA models across clinical groups
