# Supplementary material for: Widely Targeted Lipidomics of Nasal Fluid Across Upper-Airway Inflammation: Phenotype-Associated Remodeling in Allergic Rhinitis and Chronic Rhinosinusitis with Nasal Polyps
Source: Int J Mol Sci. 2026 Oct 29;27(21):9715. doi: 10.3390/ijms27219715 (PMC13763025; doi:10.3390/ijms27219715)
Supplement: Supplementary file 1 [file ijms-27-09715-s001.zip › Supplementary_Figure_S4_Kmeans_selection.pdf]

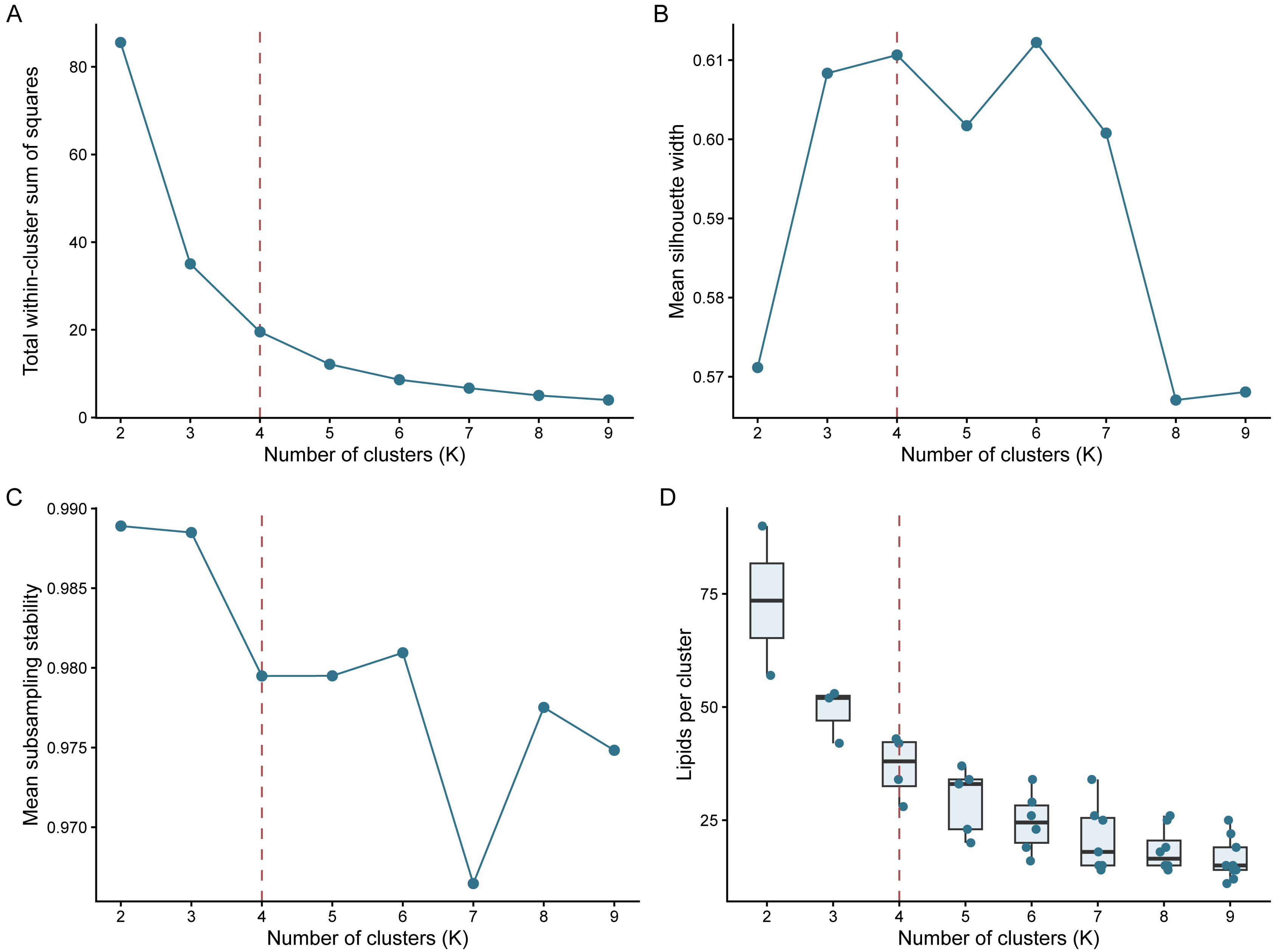

Dashed lines indicate the retained K = 4 solution; 147 lipids; 200 subsampling repeats.

Supplementary Figure S4. Selection and stability assessment of the four-module K-means clustering solution
